# Determinants of potentially harmful traditional cord care practices among mothers in Ethiopia

**DOI:** 10.3389/fped.2022.925638

**Published:** 2022-08-30

**Authors:** Bedasa Taye Merga, Gelana Fekadu, Temam Beshir Raru, Galana Mamo Ayana, Fila Ahmed Hassen, Miressa Bekana, Belay Negash, Bajrond Eshetu, Abdi Birhanu, Gutema Mulatu, Bikila Balis

**Affiliations:** ^1^School of Public Health, College of Health and Medical Sciences, Haramaya University, Harar, Ethiopia; ^2^School of Nursing and Midwifery, College of Health and Medical Sciences, Haramaya University, Harar, Ethiopia; ^3^School of Medicine, College of Health and Medical Sciences, Haramaya University, Harar, Ethiopia; ^4^Department of Environmental Health Sciences, College of Health and Medical Sciences, Haramaya University, Harar, Ethiopia

**Keywords:** umbilicus cord care, harmful practice, DHS, Ethiopia, pediatric

## Abstract

**Background:**

Globally, newborn deaths have declined from 5 million in 1990 to 2.4 million in 2019; however, the risk of death in the first 28 days is high. Harmful umbilical cord care contributes to neonatal infection, which accounts for millions of neonatal deaths. This study assessed determinants of potentially harmful traditional cord care practices in Ethiopia using data from a nationally representative survey.

**Materials and methods:**

Secondary data analyses were employed using data from the 2016 Ethiopian Demographic and Health Survey. Weighted samples of 4,402 mothers who gave birth in the last 3 years prior to the survey were included in the analysis. Binary logistic regression was fitted to identify associations of outcome variables with explanatory variable analysis, and the results were presented with an adjusted odds ratio (AOR) at a 95% confidence interval (CI), declaring statistical significance at a *p*-value < 0.05 in all analyses.

**Results:**

About 13.70% (95% CI: 12.7%, 14.7%) of mothers practice harmful traditional umbilical cord care. Maternal age (25–34 years, AOR = 1.77, 95% CI: 1.36, 2.31, 35–49 years, AOR = 1.53, 95% CI: 1.07, 2.19), maternal education (primary: AOR = 0.54, 95% CI: 0.41, 0.70 and secondary and above: AOR = 0.61, 95% CI: 0.40, 0.94), parity (para two, AOR = 0.71, 95% CI: 0.55, 0.92), and place of delivery (home delivery, AOR = 1.96, 95% CI: 1.51, 2.56) were factors associated with potentially harmful traditional umbilical cord care practices.

**Conclusion:**

Maternal educational status, parity, maternal age, and place of delivery were associated with harmful traditional cord care practices. Thus, improving mothers’ education, strengthening antenatal and postnatal care (PNC), and utilization of institutional delivery would help to reduce harmful traditional cord care practices.

## Introduction

Globally, the number of newborn deaths has declined from 5 million in 1990 to 2.4 million in 2019, however, children face the greatest risk of death in their first 28 days (1). Harmful umbilical cord care practices can directly contribute to neonatal infection, which accounts for millions of annual neonatal deaths (2, 3). Neonatal sepsis and mortality remain high in developing countries where a harmful cord care practice is high (2, 4). Africa accounts for more than 79% of the total burden of neonatal deaths worldwide (5), with neonatal deaths of 27/1,000 live births (1, 4).

Ethiopia has a relatively high neonatal death and under-5 death scores despite striving for sustainable development goals, which calls for ending preventable deaths of newborn babies and children younger than 5 years by 2030 (6). In 2019, Ethiopia ranked fourth among the countries with high neonatal mortality next to India, Nigeria, and Pakistan (1). Neonatal mortality rate in Ethiopia is 33 deaths per 1,000 live births (7). Ethiopia needs effective health measures to improve neonatal mortality to achieve the sustainable development goal (SDG) target of 12 deaths per 1,000 live birth (6).

In Ethiopia, two-thirds (66.7%) of the babies are born at home (8). In Ethiopia, severe infection is one of the top three causes of newborn death (9). National Strategy for Newborn and Child Survival has identified chlorhexidine (7.1% chlorhexidine digluconate aqueous solution or gel, delivering 4% chlorhexidine) for cord care as one of the high impact interventions to reduce the high neonatal mortality. The use of chlorhexidine, especially in setting with poor hygiene, is set as a crucial strategy to prevent life-threatening sepsis and cord infections and to avert preventable neonatal deaths in Ethiopia (10).

A neonate’s umbilical cord care is crucial in the early stage of life, and poor umbilical cord care practices have been related to infections (11). Cord infection may be confined to the umbilical cord (Omphalitis) or enter the bloodstream and become systemic (12). With standard care, the cord usually falls off between 5 and 15 days after birth. Where clean cord care is not practiced, the cord is colonized and infected by pathogenic organisms (13).

Regardless of whether the birth has taken place at home or at the health facility, substances are applied to the cord stump of the neonate; if the delivery occurred at the health facility, a substance would be placed on the cord when the infant was brought home (10). Studies from Nepal and India showed that the application of substances on the cord stump of the neonate is significantly correlated with an increased risk of omphalitis and neonatal sepsis (14, 15).

The World Health Organization (WHO) recommends the daily application of 4% chlorhexidine (7.1% chlorhexidine digluconate aqueous solution or gel, delivering 4% chlorhexidine) to the umbilical cord stump in the first week after birth only in settings where harmful traditional substances (e.g., animal dung) are commonly used on the umbilical cord (16).

However, many pieces of evidence from Ethiopia and other low-and middle-income countries have shown that mothers apply substances, such as butter, hot fermentation, lantern wax, fish bone, toothpaste, ash, charcoal, oils, vaseline, saliva, red sand, menthol-containing balm, traditional medicine, and cow dung (3, 11, 17–25). These practices often take place under unsterilized conditions; hence, they increase the risk of umbilical infection and neonatal tetanus. In Ethiopia, there is no study done on a national level on harmful traditional cord care practices. The study aimed to determine the predictors of potential harmful cord care practices among mothers in Ethiopia.

## Materials and methods

### Study setting

This study was conducted in Ethiopia, which is located in the horn of Africa. The country has nine regions [Afar; Tigray; Amhara; Oromia; Somali; Southern Nations, Nationalities, and People’s Region (SNNPR); Benishangul-Gumuz; Gambella; and Harari] and two administrative cities (Addis Ababa and Dire Dawa).

### Data sources

The study utilized data extracted from the 2016 Ethiopian Demographic and Health Survey (EDHS). Ethiopian Demographic and Health Survey collected data on basic health, demographic, and socioeconomic indicators across the nine administrative regions and two city administrations. The survey was conducted by the Central Statistical Agency (CSA) together with the Ministry of Health (MoH) and the Ethiopian Public Health Institute. The United States Agency for International Development (USAID) funded the survey. The data collection period was from 18 January 2016 to 27 June 2016 (26).

### Population and sample size

A total of 16,583 eligible women were included in the survey, 15,683 women (15–49 years) completed the interview (26). We extracted data of all mothers who gave birth in the last 3 years prior to the survey. All mothers who gave birth to alive neonates were included. Weighted samples of 4,402 mothers were included in the final analysis. Details about the DHS sampling techniques and sample size are available at http://www.dhsprogram.com/.

### Study variables

#### Dependent variable

Any substances applied to the cord except for 4% of chlorhexidine are considered potentially harmful substances (16).

#### Independent variables

This study included sex of the child (male and female), maternal education (no education, primary education, secondary, and higher education), age of the mother (15–24, 25–34, and 35–49 years), place of residence (urban and rural), and employment status (employed and unemployed), the number of the antenatal clinic (ANC) visits was also categorized into no ANC visits, 1–3 ANC visits, four or more ANC visits, and places of delivery (categorized as a health facility or home). Parity was also categorized into para one, para two, para three, or more.

In this study, the 11 regions of Ethiopia were categorized into three contextual regions: pastoralist, agrarian, and city (which were defined on the basis of the socioeconomic and cultural backgrounds of their populations) (27).

Wealth indexes (categorized as poorest, poorer, middle, richer, and richest) were used to indicate a household’s wealth status. The wealth index was constructed using data on a household’s ownership of selected assets, such as television and bicycles, materials used for housing construction, and types of water access and sanitation facilities (26).

### Statistical analysis

Analyses were performed using STATA version 14. Descriptive statistics, such as frequency and proportion, were used to describe the characteristics of the data. To assess the association between explanatory variables and potentially harmful umbilical cord care practices of mothers with children aged 0–12 months, a binary logistic regression model was fitted. First, each variable was entered into a binary logistic regression model. Second, variables, which were significant at a *p*-value of less than or equal to 0.25, were fitted into a multivariable logistic regression model to identify independent factors of harmful umbilical cord care practices among mothers with children aged 0–12 months in Ethiopia. Statistical significance was declared at a *p*-value < 0.05 in all analyses. The results from the logistic regression analyses are presented as adjusted odds ratios (AORs) with 95% confidence intervals (CIs).

## Results

### Sociodemographic characteristics of study participants

A total of 4,402 mothers were included in the analysis. More than half (52.94%) of the mothers were in the 25–34 years of age group. Regarding the religion of the mothers, Muslim followers account for a major part (48.47%) followed by Orthodox (29.79%). Three-fifths (63.94%) of the respondents had no formal education, and 88.76% were from the agrarian region. The ratio of male to female children was almost 1:1. Urban resident accounts for majority of the respondents (89.99%). One out of four (25.28%) was found in the poorest wealth index (Table 1).

**TABLE 1:** Sociodemographic characteristics of mothers in Ethiopia, 2016.

Variables	Frequency	Percent
Age		
15–29	1,228	27.89
25–34	2,330	52.94
35–49	844	19.17
Religion		
Orthodox	1,311	29.79
Protestant	799	18.15
Muslim	2,134	48.47
Other*	158	3.58
Educational level		
No education	2,815	63.94
Primary	1,277	29.02
Secondary and higher	310	7.04
Economic regions		
Agrarians	3,907	88.76
Pastoralists	380	8.64
City dwellers	115	2.60
Child sex		
Boy	2,231	50.68
Girl	2,171	49.32
Place of residence		
Urban	441	10.01
Rural	3,961	89.99
Wealth index		
Poorest	1,113	25.28
Poorer	1,074	24.40
Middle	882	20.02
Richer	749	17.02
Richest	584	13.27

*Catholic and traditional.

### Obstetrics history

Almost one out of three (33.96%) mothers received all ANC visits whereas an equal proportion did not take ANC visits. Regarding mode of delivery, the majority (97.78%) gave spontaneous vaginal delivery and 71.80% gave birth at home (Table 2).

**TABLE 2:** Obstetric history of postnatal mothers in Ethiopia, 2016.

Variables	Frequency	Percent
Antenatal follow-up		
No visits	837	32.91
1–3 visits	843	33.13
Four and more visits	864	33.96
Mode of delivery		
Spontaneous vaginal delivery	4,304	97.78
Cesarean sections	98	2.22
Parity		
Para 1	1,075	24.41
Para 2	2,043	46.40
Para ≥ 3	1,284	29.18
Place of delivery		
Home	3,161	71.80
Facility	1,241	28.20

### Harmful traditional newborn umbilicus cord care practice

In this study, 13.70% (95% CI: 12.7%, 14.7%) of mothers applied non-medically approved substances on newborns’ umbilical stump. Of the total substances they applied, the most common were any type of oil (72.70%), ointments (17.09%), ash (1.97%), and dung (0.29%; Table 3).

**TABLE 3:** Potentially harmful traditional umbilicus cord care practice among postnatal mothers in Ethiopia, 2016.

Variables	Frequency	Percent
Application to umbilicus		
Yes	603	13.70
No	3,799	86.30
Any type of oil (*n* = 603)		
No	165	27.30
Yes	438	72.70
Dung (*n* = 603)		
No	601	99.71
Yes	2	0.29
Ash (*n* = 603)		
No	591	98.03
Yes	12	1.97
Ointment (*n* = 603)		
No	500	82.91
Yes	103	17.09
Unknown material (*n* = 603)		
No	549	91.02
Yes	54	8.98

### Predictors of potentially harmful traditional cord care practices

In bivariate logistic regression, place of residence, maternal age, maternal educational level, place of delivery, parity, wealth index, and ANC visits were factors significantly associated with potentially harmful cord care practices.

However, in multiple logistic regressions, maternal age ranging from 25 to 34 years was 1.77 times (AOR: 1.77, 95% CI: 1.36, 2.31) and age ranging from 35 to 49 was 1.53 times (AOR: 1.53, 95% CI: 1.07, 2.19) more likely to be associated with harmful traditional cord care practices. In contrast, mothers who had primary education were 0.54 times (AOR: 0.54, 95% CI: 0.41, 0.70) and secondary and above educational levels were 0.61 times (AOR: 0.61, 95% CI: 0.40, 0.94) less likely to be associated with harmful traditional cord care practices, respectively. Similarly, para two mothers were less likely to be associated with harmful traditional cord care practices as compared to mothers with less than two para (AOR: 0.71, 95% CI: 0.55, 0.92). Mothers who gave birth to their last child at home were almost two times more likely to be associated with harmful traditional cord care practices (AOR: 1.96, 95% CI: 1.51, 2.56) (Table 4).

**TABLE 4:** Predictors of potentially harmful traditional cord care practices among postnatal mothers in Ethiopia, 2016.

Variables	COR (95% CI)	*P*-value	AOR (95% CI)	*P*-value
Place of residence				
Urban	1		1	
Rural	1.42 (1.10, 1.85)	0.008	1.01 (0.63, 1.60)	0.989
Age				
15–24	1		1	
25–34	2.01 (1.75, 2.55)	0.000	1.77 (1.36, 2.31)	0.000
35–49	2.33 (1.80, 3.04)	0.000	1.53 (1.07, 2.19)	0.018
Sex of the child				
Boy	1		1	
Girl	1.18 (0.99, 1.40)	0.063	1.11 (0.89, 1.39)	0.334
Education level				
No education	1		1	
Primary	0.44 (0.37, 0.53)	0.000	0.54 (0.41, 0.70)	0.000
Secondary and higher	0.41 (0.31, 0.56)	0.000	0.61 (0.40, 0.94)	0.024
Parity				
Para 1	1		1	
Para 2	1.07 (0.87, 1.32)	0.50	0.71 (0.55, 0.92)	0.010
Para ≥ 3	1.41 (1.11, 1.80)	0.005	0.85 (0.60, 1.20)	0.357
Place of delivery				
Health facility	1		1	
Home	2.41 (2.02, 2.87)	0.000	1.96 (1.51, 2.56)	0.000
Economic regions				
Pastoralists	1		1	
Agrarians	1.04 (0.77, 1.41)	0.801	0.93 (0.59, 1.47)	0.770
City dwellers	1.43 (0.74, 2.79)	0.288	2.17 (0.94, 5.05)	0.071
Wealth index				
Richest	1		1	
Poorest	1.37 (1.05, 1.80)	0.021	0.73 (0.45, 1.17)	0.194
Poorer	1.46 (1.11, 1.92)	0.007	1.14 (0.71, 1.83)	0.574
Middle	1.76 (1.31, 2.36)	0.000	1.02 (0.64, 1.64)	0.919
Richer	1.46 (1.09, 1.97)	0.012	1.21 (0.76, 1.92)	0.429
ANC visits				
No ANC Visits	1		1	
1–3 visits	0.7 (0.53, 0.93)	0.013	0.92 (0.67, 1.24)	0.579
Four and more visits	0.54 (0.41, 0.70)	0.000	0.76 (0.55, 1.04)	0.082
Employment				
Unemployed	1		1	
Employed	1.02 (0.86, 1.22)	0.79	1.04 (0.82, 1.31)	0.752

COR, crude odds ratio; AOR, adjusted odds ratio; CI, confidence interval.

## Discussion

This population-based cross-sectional survey provides factual insights regarding the determinants of potentially harmful traditional cord care practices in Ethiopia. This study revealed that more than one in eight mothers applied substances not medically recommended, which potentially increases the risk of cord infection. The practices of applying traditional substances to the cord stump in Ethiopia call for actions that increase antenatal care, delivery services, and postnatal care (PNC) utilizations where both mothers and newborns are benefited. Clean, dry cord care is recommended by WHO for newborns in health facilities and at home in low neonatal mortality settings. The use of chlorhexidine in these situations may be considered only to replace the application of a harmful traditional substances, such as cow dung to the cord stump (16).

In this study, 13.70% of mothers applied potentially harmful substances, such as dung, oil, ointments, ash, and butter on the umbilical stump of the neonates. This finding was lower than the studies done in Benin City (46.1%) (17), Nigeria (22.2 and 67.3%) (18, 19), Nepal (26 and 43%) (28), Uganda 39.6% (3), Southwest Ethiopia (20.4 and 54.7%) (20, 23), and Eastern Ethiopia (29.3%) (24). The discrepancy may be due to variations in access to awareness, geographical variations, and health-seeking behavior across the different cultural beliefs. This has health implications for newborns’ infection and tetanus (29–32).

The maternal ages ranging from 25 to 34 and from 35 to 49 years were positively associated with harmful traditional cord care practices as compared to the maternal age ranging from 15 to 24 years. This is supported by a study done in Nigeria (18). The possible justification might be that the older mothers were less likely to adhere to ANC visits, skilled birth attendants, and PNC visits, which help them to get counseling from care providers on recommended cord care practices. In addition, older mothers learn or are influenced by society who might not always be the source of the correct information and the belief that applying substances could help facilitate fast healing (19, 20).

Mothers with primary education and secondary/above had lower odds of application of potentially harmful substances to the stump of the umbilical cord when compared with mothers with no formal education. These findings are consistent with studies that show the positive associations between a higher level of maternal education and better health-seeking behavior and thus medically recommended child cord care practices (7, 20).

Similarly, in our study, we found that mothers who gave birth two times revealed a large reduction in the odds of practicing potentially harmful traditional cord care. This is due to the fact that mothers who have exposure to childbirth have a chance to get clean cord care information from health care providers (33–36). This can be explained by mothers having previous experience of delivery and obtaining information about essential newborn care during their previous ANC, delivery, PNC, and immunization periods, and this can inspire mothers to practice essential newborn care more.

Mothers who delivered their last child at home had higher odds of practicing potentially harmful traditional cord care as compared to those who delivered at a health institution. The reason for this could be the fact that mothers who gave birth at home may be influenced by social customs or were unaware of the health risks involved related to the application of potentially harmful substances to the stump of the umbilical cord (11, 37). When the birth is at the relatives, traditional birth attendants or neighbors may apply substances to the umbilicus of the newborn (34). In contrast, mothers who gave birth at the health facility were more likely to receive ANC, which creates an opportunity to counsel mothers about recommended newborn cord care practices and to be aware of risks of infection related to the application of harmful substances on the stump of the umbilical cord (28, 38). This implies that pregnant women need ANC, skilled birth attendants, and postpartum care to promote better newborn care practices, specifically hygienic cord care.

### Strengths and limitations

The study utilized data from a nationally representative survey, which could be considered a strength. However, this study was not without limitations. Harmful traditional substances applied on the umbilical stump of the newborns were a self-reported event by the mother/caregiver, which may result in recall bias. The data provided the proportion of women who applied anything to the cord stump and some substances, which are potentially harmful; however, the data did not present the proportion of 4% chlorhexidine applied to the stump, which is recommended by WHO. The other limitation is that the cross-sectional study design cannot establish a temporal relationship between the outcome and response variables.

## Conclusion

A significant proportion of women practice harmful traditional cord care in Ethiopia, and multifaceted factors appear to determine their practices. Maternal educational status, parity, maternal age, and place of delivery were independently associated with harmful traditional cord care practices. Thus, intervention strategies that emphasize primipara and older women to practice safe umbilical cord care may halt the problem. Moreover, improving the educational status of women and utilization of institutional delivery would help to reduce the prevalence of harmful traditional cord care practices.

## Data availability statement

Publicly available datasets were analyzed in this study. This data can be found here: The datasets used for analysis are available from http://www.dhsprogram.com/ up on reasonable request.

## Ethics statement

The study was ethically reviewed and approved by the National Research Ethics Review Committee (NRERC) of Ethiopia. Written informed consent was obtained from participants prior to data collection.

## Author contributions

BB and BM: conceived and designed the study. BM, TR, GA, and GF: methodology. MB, FH, BE, and BN: analysis. BM, AB, GM, and BB: drafting the manuscript and made revisions. All authors contributed to data analysis, drafting, or revision of the article, gave final approval of the version to be published, and agreed to be accountable for all aspects of the work.

## Abbreviations

AOR: adjusted odds ratio
ANC: antenatal care
COR: crude odds ratio
PNC: postnatal care.
